# Racial and Ethnic Differences in Hospice Use Among Medicaid-Only and Dual-Eligible Decedents

**DOI:** 10.1001/jamahealthforum.2023.4240

**Published:** 2023-12-08

**Authors:** Julie Robison, Noreen Shugrue, Ellis Dillon, Deborah Migneault, Doreek Charles, Dorothy Wakefield, Bradley Richards

**Affiliations:** 1 UConn Health, Center on Aging, Farmington, Connecticut; 2 Connecticut Department of Social Services, Hartford, Connecticut; 3 Yale School of Medicine, New Haven, Connecticut; 4 Yale School of Management, New Haven, Connecticut

## Abstract

**Importance:**

Hospice care enhances quality of life for people with terminal illness and is most beneficial with longer length of stay (LOS). Most hospice research focuses on the Medicare-insured population. Little is known about hospice use for the racially and ethnically diverse, low-income Medicaid population.

**Objective:**

To compare hospice use and hospice LOS by race and ethnicity among Medicaid-only individuals and those with dual eligibility for Medicare and Medicaid (duals) in the Connecticut Medicaid program who died over a 4-year period.

**Design, Setting, and Participants:**

This retrospective population-based cohort study used Medicaid and traditional Medicare enrollment and claims data for 2015 to 2020. The study included Connecticut Medicaid recipients with at least 1 of 5 most common hospice diagnoses who died from 2017 to 2020.

**Exposure:**

Race and ethnicity.

**Main Outcomes and Measures:**

Hospice use (yes/no) and hospice LOS (1-7 days vs ≥8 days.) Covariates included sex, age, and nursing facility stay within 60 days of death.

**Results:**

Overall, 2407 and 23 857 duals were included. Medicaid-only decedents were younger (13.8% ≥85 vs 52.5%), more likely to be male (50.6% vs 36.4%), more racially and ethnically diverse (48.7% non-Hispanic White vs 79.9%), and less likely to have a nursing facility stay (34.9% vs 56.1%). Race and ethnicity were significantly associated with hospice use and LOS in both populations: non-Hispanic Black and Hispanic decedents had lower odds of using hospice than non-Hispanic White decedents, and Hispanic decedents had higher odds of a short LOS. In both populations, older age and female sex were also associated with more hospice use. For duals only, higher age was associated with lower odds of short LOS. For decedents with nursing facility stays, compared with those without, Medicaid-only decedents had higher odds of using hospice (odds ratio [OR], 1.49; 95% CI, 1.24-1.78); duals had lower odds (OR, 0.60; 95% CI, 0.57-0.63). Compared with decedents without nursing facility stays, duals with a nursing facility stay had higher odds of short LOS (OR, 2.63; 95% CI, 2.43-2.85).

**Conclusions and Relevance:**

Findings raise concerns about equity and timing of access to hospice for Hispanic and non-Hispanic Black individuals in these understudied Medicaid populations. Knowledge about, access to, and acceptance of hospice may be lacking for these low-income individuals. Further research is needed to understand barriers to and facilitators of hospice use for people with nursing facility stays.

## Key Points

**Question:**

What is the prevalence of hospice use and short hospice stay by race and ethnicity in the low-income Medicaid-only and dual-eligible populations?

**Findings:**

In this cohort study, in both Medicaid only and dual-eligible populations, Hispanic and non-Hispanic Black individuals had the lowest odds of receiving hospice, and Hispanic individuals had the highest odds of a short hospice stay; there were also differences for individuals with a nursing facility stay.

**Meaning:**

These findings raise concerns about equity and timing of access to hospice for both Medicaid populations, particularly for Hispanic and non-Hispanic Black decedents, and suggest further analysis of the role of nursing facility stays.

## Teaser

This cohort study compares hospice use and length of stay by race and ethnicity among Medicaid-only and dual-eligible individuals in Connecticut’s Medicaid program who died between 2017 to 2020 with 1 of the 5 most common diagnoses for hospice decedents.

## Introduction

Concerns about disparities in end-of-life care have gained momentum owing to health inequities observed during the COVID-19 pandemic. However, a lack of available data outside of Medicare about hospice use has made assessing these disparities challenging.^1^ Although Medicare beneficiaries make up the largest and most studied population of hospice recipients, hospice is also covered by commercial insurance and state Medicaid programs.^2^ However, most hospice research has focused only on the Medicare-insured population. Numerous studies have explored differences in hospice use and hospice length of stay (LOS) for Medicare recipients by factors including race and ethnicity.^3,4^ Although more than half of White Medicare decedents received hospice in 2020, compared with only a third of Hispanic, non-Hispanic Black, and other racial and ethnic groups,^4^ the results of studies examining hospice use and LOS by race and ethnicity are conflicting.^3,5,6,7^ Moreover, little is known about those differences for the racially and ethnically diverse, low-income individuals with Medicaid. Two studies^5,8^ suggested hospice underuse by Medicaid-insured patients aged 21 to 64 years with advanced cancer.

Although eligibility for Medicaid varies by state, all recipients have low income. Medicaid-insured individuals may have either Medicaid only (having Medicaid as their only source of health insurance), or be dual eligible (qualifying for both Medicaid and Medicare by virtue of age or disability). Demographics of the 2 populations differ substantially by age and race and ethnicity. Because most people ages 65 years or older qualify for Medicare (exceptions relate to citizenship/immigration status or lack of Medicare-covered employment), the Medicaid-only population is younger and has higher percentages of Hispanic and non-Hispanic Black individuals.^9^

Hospice benefits are provided to people with life-limiting illnesses, and include medical care, pain and symptom management, and emotional and spiritual support.^4^ Medicare enacted the Medicare hospice benefit (MHB) in 1982^10^ for individuals with a life expectancy of 6 months or fewer who agree to forgo curative care.^4,11^ MHB use grew rapidly to over 50% of decedents in 2015,^12^ and remains at approximately half.^1,13^ Most state Medicaid programs adopted hospice benefits similar to the MHB,^1,14^ but hospice use for the Medicaid-only population has not been systematically examined to our knowledge. Although cancer diagnoses originally predominated for MHB recipients, most people enrolling in hospice now have noncancer diagnoses.^4^ Nevertheless, hospice remains highly underused.^15^ The 5 most common diagnoses for Medicare hospice decedents in 2020 were dementia, heart disease, cancer, respiratory illness, and stroke.^4^ Hospice care is most beneficial when LOS is longer: LOS of 1 to 7 days is considered a marker of poor quality care and patient dissatisfaction.^16^ Longer stays improve quality of life and symptom control,^17^ whereas late entry results in lower satisfaction and greater unmet need.^18^ Despite extensive evidence of the benefits of longer stays, in 2020 half of MHB recipients were enrolled for 18 or fewer days, and a quarter for only 1 to 5 days.^4^

Evidence for the role of race and ethnicity in explaining differences in hospice use is mixed. A systematic review^3^ of 1980 to 2006 literature on racial and ethnic disparities in hospice care found disparities in hospice use among African American patients compared with White patients in 12 of 13 studies, but lacked power to make conclusions about other racial and ethnic groups and included primarily people with Medicare and cancer diagnoses. Some more recent studies^5,6,7^ found no significant racial and ethnic disparities in hospice use. Others did find racial or ethnic differences, including that Black and Hispanic individuals with advanced cancer had lower odds of receiving a hospice referral,^19^ and that Black individuals were less likely to use hospice for 3 or more days.^20^ Although Yang et al^5^ found low hospice use in a small sample of New Jersey Medicaid recipients with advanced cancer, they observed no significant differences in hospice use by race and ethnicity. Although these mixed results may be due to different data sources, metrics, or populations, they emphasize the importance of investigating the quality of end-of-life care among Medicaid recipients and racial and ethnic groups.

This study compared hospice use and LOS by race and ethnicity among Medicaid-only and dual-eligible individuals (henceforth duals) in Connecticut’s Medicaid program who died between 2017 and 2020 with 1 of the 5 most common diagnoses for hospice decedents. Connecticut’s Medicaid hospice benefit has the same eligibility criteria, services, and payment rates as the MHB.

## Methods

This study followed the Strengthening the Reporting of Observational Studies in Epidemiology (STROBE) reporting guidelines for cohort studies.

### Study Design and Sample

Data came from Medicaid and traditional Medicare claims data for Connecticut Medicaid recipients who died between 2017 and 2020. The sample was drawn from an integrated data set of Medicaid enrollment and claims data from the Connecticut Medicaid Management Information System and federal Medicare Part A, B, D enrollment and Chronic Conditions Warehouse (CCW) data. Because the analyses examined hospice claims in the year before death, the sample included only duals who had continuous traditional Medicare coverage in the year before death. It was determined that this retrospective analysis of claims data did not meet UConn Health institutional review board criteria for human participants research; therefore, no written informed consent or waiver were required.

Because Medicaid-only decedents are significantly younger on average than duals, and younger populations experience more causes of death unlikely to be appropriate for hospice care (eg, unintentional injury, suicide, or homicide),^21^ we restricted the sample to individuals who had at least 1 of the 5 most common diagnoses of hospice decedents in the 2 years prior to death: cancer (breast, colon, lung, pancreatic, prostate), heart disease (congestive heart failure, acute myocardial infarction, ischemic heart attack), dementia, respiratory illness (chronic obstructive pulmonary disease), and stroke.^4^ We used CCW data to identify all diagnoses except pancreatic cancer, which is not included in the CCW. We used Medicare or Medicaid claims data to identify a diagnosis of pancreatic cancer in the 2 years prior to death to align with the CCW reference period for other cancers.

A total of 35 086 Medicaid recipients died during 2017 to 2020 and, for duals, had at least 12 months of traditional Medicare enrollment prior to death. Of these, 26 283 recipients (74.9%) had at least 1 of the eligible diagnoses in the 2 years before death. Nineteen people were excluded due to missing race and ethnicity data, leaving an analytic sample of 26 264.

### Measures

Medicaid-only and dual populations were analyzed separately. Using enrollment data, decedents were categorized as duals if they were eligible for both Medicare and Medicaid at any time during the 12 months prior to death; otherwise, they were categorized as Medicaid only. Outcome measures include any hospice use (yes/no) and hospice LOS (1-7 days vs ≥8 days). We analyzed Medicare and Medicaid claims data to identify if a decedent had any hospice use during the year prior to death. Using claims dates of service, we identified all episodes of hospice use and calculated the total number of days per episode. If there was a gap of 7 or fewer days between episodes, they were combined and counted as a single episode of hospice use. Using only the hospice episode closest to death, we constructed a variable calculating the number of hospice days the decedent used and dichotomized the variable into 1 to 7 days or 8 or more days of hospice use.

Independent variables included race and ethnicity, sex, age at time of death, and nursing facility residence within 60 days of death. Researchers continue to face challenges with the completeness and accuracy of race and ethnicity data. To facilitate complete race and ethnicity variables, we matched our sample to multiple data sources (home health [OASIS] and nursing facility [MDS] assessments, Medicare and Medicaid claims, and Connecticut Universal Assessment data). If race and ethnicity data were missing or marked as other in the integrated data set, we used a step-by-step approach with the state’s Universal Assessment as the primary data source, OASIS data second, then MDS, Medicaid claims, finally Medicare claims. Racial and ethnic identification in some sources was made by the individuals and in some by case workers. A race and ethnicity variable was constructed with 5 categories: Hispanic, non-Hispanic Asian, non-Hispanic Black, non-Hispanic White, and other. The other category included Native American individuals and persons directly classified as other by individuals or case workers. Although Medicare generally does not pay for nursing facility custodial care, Medicaid is the leading payer of such care nationally.^1^ Because most hospice research concerns only the Medicare population, the effect of nursing facility stays on hospice use for Medicaid recipients is not well understood. Nursing facility stays were identified using Medicare and Medicaid claims data. We created a variable to identify decedents who were nursing facility residents within 60 days of death (yes/no).

### Analysis

Frequencies and percentages were calculated for each variable. Within the Medicaid-only and dual populations, χ^2^ analyses compared sample characteristics (race and ethnicity, age, sex, diagnoses, and nursing facility stay within 60 days of death) by hospice use. Separate logistic regression models for the Medicaid-only and dual populations were used to examine the associations between hospice use and hospice LOS and decedent characteristics. Models included race and ethnicity, age, sex, and nursing facility stay within 60 days of death. Outcome variables were any hospice use (yes/no) and short hospice LOS (1-7 vs ≥8 days) for each population. Statistical analysis was performed using SAS statistical software (version 9.4, SAS Institute). A *P* value <.05 indicated statistical significance. Final analyses were conducted in September 2023.

## Results

Table 1 and Table 2 display sample characteristics for the Medicaid-only and dual populations, respectively, and provide descriptive details comparing hospice recipients and nonrecipients. For both the Medicaid-only and dual populations, a higher percentage of people who received hospice compared with those without hospice care, were non-Hispanic White, older, female, and people diagnosed with cancer or dementia. Comparing across Table 1 and Table 2, the Medicaid-only population was smaller, more racially and ethnically diverse than the dual population (non-Hispanic White: 1172 [48.7%] vs 19 062 [79.9%]), younger, had a lower proportion of female decedents (1189 [49.4%] vs 15 183 [63.6%]), and received hospice less frequently (773 [32.1%] vs 11 595 [48.6%]). Although everyone in both populations had at least 1 qualifying diagnosis, duals were more likely to have multiple qualifying diagnoses (14 697 [61.6%] vs 639 [26.5%]). Only 842 Medicaid-only decedents (34.9%) had a nursing facility stay within 60 days of death vs 13 385 duals (56.1%). Approximately 40% of both groups had a short hospice LOS.

**Table 1.** Sample Characteristics Among 2407 Medicaid-Only Participants^a^

Characteristic	No. (%)		
	Total (N = 2407)	Received hospice (N = 773)	Did not receive hospice (N = 1634)
Race and ethnicity^b^			
Hispanic	488 (20.3)	143 (18.5)	345 (21.2)
Non-Hispanic Asian	64 (2.7)	29 (3.8)	35 (2.1)
Non-Hispanic Black	430 (17.9)	125 (16.2)	305 (18.7)
Non-Hispanic White	1172 (48.7)	412 (53.3)	760 (46.5)
Other^c^	253 (10.5)	64 (8.3)	189 (7.9)
Age, y^b^			
≤44	156 (6.5)	24 (3.1)	132 (8.1)
45-54	395 (16.4)	100 (12.9)	295 (18.1)
55-64	937 (38.9)	288 (37.3)	649 (39.7)
65-74	351 (14.6)	119 (15.4)	232 (14.2)
75-84	235 (9.8)	85 (11.0)	150 (9.2)
≥85	333 (13.8)	157 (20.3)	176 (10.8)
Female^b^	1189 (49.4)	437 (56.5)	752 (46.0)
Male	218 (50.6)	336 (43.5)	882 (54.0)
Qualifying diagnoses			
Cancer^b^	149 (6.1)	76 (9.8)	73 (4.5)
Dementia^b^	878 (36.5)	386 (49.9)	492 (30.1)
Heart disease^b^	1378 (57.3)	355 (45.9)	1023 (62.6)
Respiratory illness^b^	355 (14.8)	88 (11.4)	267 (16.3)
Stroke	382 (15.9)	128 (16.6)	254 (15.5)
No. of qualifying diagnoses (5 possible)			
1	1768 (73.5)	550 (71.2)	1218 (74.5)
2	550 (22.9)	190 (24.6)	360 (22.0)
≥3	89 (3.7)	33 (4.3)	56 (3.4)
In a nursing facility within 60 d before death^b^	842 (34.9)	330 (42.7)	512 (31.3)
Short hospice LOS (≤7 d)	NA	320 (41.4)	NA

Abbreviations: LOS, length of stay; NA, not applicable.

^a^ Significance tests are between the received hospice and did not receive hospice columns.

^b^ *P* ≤ .001.

^c^ Other includes Native American and people classified as other by self or case workers.

**Table 2.** Sample Characteristics Among 23 875 Dual-Eligible Participants^a^

Characteristic	No. (%)		
	Total (N = 23 857)	Received hospice (N = 11 595)	Did not receive hospice (N = 12 262)
Race and ethnicity^b^			
Hispanic	1853 (7.8)	856 (7.4)	997 (8.1)
Non-Hispanic Asian	415 (1.7)	149 (1.3)	266 (2.2)
Non-Hispanic Black	2405 (10.1)	953 (8.2)	1452 (11.8)
Non-Hispanic White	19 062 (79.9)	9582 (82.6)	9480 (77.3)
Other^c^	122 (0.5)	55 (0.5)	67 (0.5)
Age, y^b^			
≤44	166 (0.7)	23 (0.2)	143 (1.2)
45-54	517 (2.2)	132 (1.1)	385 (3.1)
55-64	1614 (6.8)	528 (4.6)	1086 (8.9)
65-74	3437 (14.4)	1361 (11.7)	2076 (16.9)
75-84	5603 (23.5)	2675 (23.1)	2928 (23.9)
≥85	12 520 (52.5)	6876 (59.3)	5644 (46.0)
Female^b^	15 183 (63.6)	7865 (67.8)	7318 (59.7)
Male	8674 (36.4)	3730 (32.2)	4944 (40.3)
Qualifying diagnoses			
Cancer^b^	2985 (12.5)	1748 (15.1)	1237 (10.1)
Dementia^b^	16 596 (69.6)	8758 (75.5)	7838 (63.9)
Heart disease^b^	16 020 (67.2)	7467 (64.4)	8553 (69.8)
Respiratory illness^c^	4714 (19.8)	2212 (19.1)	2502 (20.4)
Stroke	4777 (20.0)	2398 (20.4)	2409 (19.6)
No. of qualifying diagnoses (5 possible)^b^			
1	9160 (38.4)	4126 (35.6)	5034 (41.1)
2	9273 (38.9)	4613 (39.8)	4660 (38.0)
3 or more	5424 (22.7)	2856 (24.6)	2568 (20.9)
In a nursing facility within 60 d before death^b^	13 385 (56.1)	5967 (51.5)	7481 (60.5)
Short hospice LOS (≤7 d)	NA	4591 (39.6)	NA

Abbreviations: LOS, length of stay; NA, not applicable.

^a^ Significance tests are between the received hospice and did not receive hospice columns.

^b^ *P* < .001.

^c^ Other includes Native American and people classified as other by self or case workers.

In bivariate analysis, race and ethnicity was significantly associated with both hospice use and short LOS for the Medicaid-only population (Figure). Individuals in the other race category, followed by non-Hispanic Black and Hispanic decedents, had the lowest use, whereas non-Hispanic White and Asian decedents had the highest. Of those who received hospice, other race, Hispanic and non-Hispanic Black decedents had the highest percentage of short LOS. For duals, race and ethnicity was significantly associated only with hospice use. Non-Hispanic White decedents received hospice more than all other racial and ethnic categories.

**Figure.**
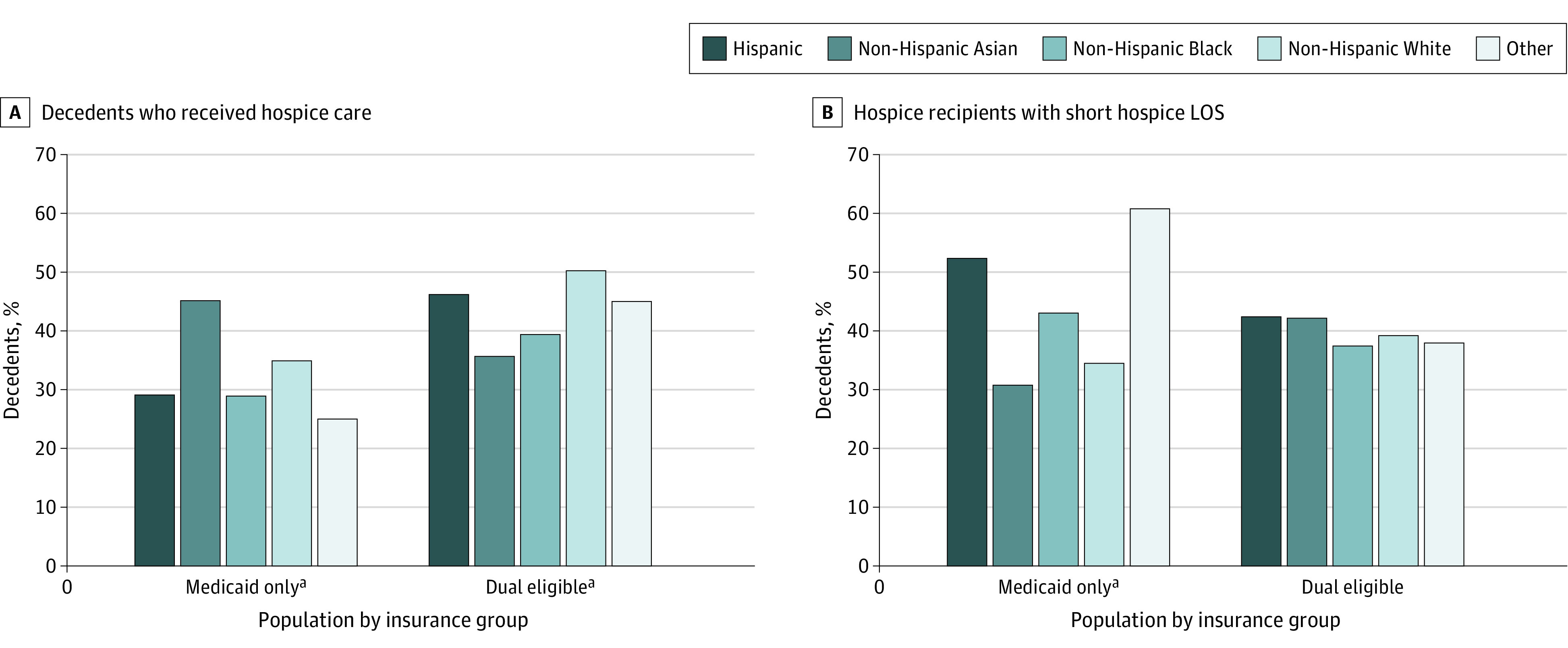
Percentage of Decedents by Race and Ethnicity Receiving Hospice and With Short Hospice Length of Stay (LOS) ^a^*P* < .001.

Separate multivariable logistic regression models for each population identified significant associations with hospice use and short hospice LOS (Table 3 and Table 4). The association of race and ethnicity with hospice use was the same for both populations: Hispanic and non-Hispanic Black individuals had lower odds of using it compared with non-Hispanic White decedents. For duals, non-Hispanic Asian individuals also had lower odds of using hospice than non-Hispanic White decedents (odds ratio [OR], 0.52; 95% CI, 0.42-0.64). Older individuals and women had higher odds of using hospice in both populations. However, results differed in the odds of using hospice for decedents who had a nursing facility stay within 60 days of death compared with those without: Medicaid-only decedents had higher odds of using hospice (OR, 1.49; 95% CI, 1.24-1.78), whereas duals had lower odds (OR, 0.60; 95% CI, 0.57-0.63). In both populations, Hispanic individuals had higher odds of a short hospice LOS than non-Hispanic White individuals and women had lower odds than men. For duals only, older individuals had lower odds of a short hospice LOS, whereas those with a nursing facility stay within 60 days of death had higher odds (OR, 2.63; 95% CI, 2.43-2.85).

**Table 3.** Logistic Regression–Medicaid Only

Independent variable	OR (95% CI)			
	Received hospice	*P* value	Short hospice LOS (≤7 d)	*P* value
Race and ethnicity^a^				
Hispanic	0.76 (0.60-0.96)	.02	2.32 (1.55-3.48)	<.001
Non-Hispanic Asian	1.32 (0.79-2.21)	.30	1.17 (0.51-2.71)	.71
Non-Hispanic Black	0.77 (0.60-0.98)	.03	1.36 (0.89-2.07)	.16
Other^b^	0.81 (0.59-1.11)	.19	2.08 (1.18-3.66)	.01
Age, y^c^				
45 to 64	2.06 (1.31-3.25)	.002	1.98 (0.83-4.72)	.13
≥65	2.78 (1.74-4.42)	<.001	0.78 (0.33-1.88)	.58
Female	1.38 (1.15-1.65)	<.001	0.71 (0.52-0.98)	.03
Male	1 [Reference]		1 [Reference]	
Nursing facility within 60 d of death	1.49 (1.24-1.78)	<.001	1.14 (.83-1.55)	.42

Abbreviations: LOS, length of stay; OR, odds ratio.

^a^ Reference group for race and ethnicity is non-Hispanic White because prior research evidence shows non-Hispanic White individuals are more likely to receive hospice care than people of other races or ethnicities.

^b^ Other includes Native American and people classified as other by self or case workers.

^c^ Reference group for Medicaid only age is 18 to 44 years.

**Table 4.** Logistic Regression–Dual Eligible

Independent variable	OR (95% CI)			
	Received hospice	*P* value	Short hospice LOS (≤7 d)	*P* value
Race and ethnicity^a^				
Hispanic	0.89 (0.80-0.98)	.02	1.25 (1.08-1.45)	.003
Non-Hispanic Asian	0.52 (0.42-0.64)	<.001	1.29 (0.92-1.81)	.14
Non-Hispanic Black	90.74 (0.68-0.81)	<.001	0.87 (0.75-1.00)	.05
Other^b^	0.81 (0.56-1.16)	.25	0.99 (0.57-1.73)	.98
Age, y^c^				
65 to 74	1.64 (1.47-1.84)	<.001	0.81 (0.67-0.98)	.03
75 to 84	2.30 (2.07-2.56)	<.001	0.74 (0.62-0.88)	<.001
≥85	3.00 (2.71-3.32)	<.001	0.66 (0.56-0.78)	<.001
Female	1.21 (1.14-1.28)	<.001	0.79 (0.72-0.86)	<.001
Male	1 [Reference]		1 [Reference]	
Nursing facility within 60 d of death	0.60 (0.57-0.63)	<.001	2.63 (2.43-2.85)	<.001

Abbreviation: LOS, length of stay.

^a^ Reference group for race and ethnicity is non-Hispanic White because prior research evidence shows non-Hispanic White individuals are more likely to receive hospice care than people of other races or ethnicities.

^b^ Other includes Native American and people classified as other by self or case workers.

^c^ Reference group for dual eligible age is younger than 65 years.

## Discussion

Hospice use by Medicare recipients has been studied extensively, but less is known about hospice use by low-income, racially and ethnically diverse Medicaid beneficiaries. This study of Medicaid-only and dual-eligible decedents with 1 of the 5 most common hospice diagnoses in 1 state over a 4-year period provides insight into how race and ethnicity and other factors are associated with hospice use and hospice LOS for these understudied populations. Because of many population differences between Medicaid-only and duals, including race and ethnicity, age, sex, and number of diagnoses, direct comparison of hospice use in the 2 populations is not meaningful. This study focused on racial and ethnic differences in hospice use and LOS within each population.

Medicaid-only results point to some disparities in both hospice use and LOS. Hispanic and non-Hispanic Black individuals used hospice significantly less often than non-Hispanic White individuals, and Hispanic individuals and people in the other race category were more than twice as likely as non-Hispanic White participants to have a short hospice LOS. These novel findings about the Medicaid-only population are consistent with the findings of this study with respect to the dual population, where similar disparities exist, and confirm some previous research^3,19,20^ despite a few contrary findings.^5,6,7^ Access to and use of hospice services may be influenced by language barriers, possibly recent immigration, and lack of understanding about hospice in the US as well as important cultural beliefs or values about the end of life for both the Hispanic population and the non-Hispanic Black population.^5,20,22,23,24^ These disparities may reflect mistrust issues about the hospice service and/or the information received from health care professionals along with potential racial biases that affect who a physician is more likely to refer to hospice.^5,20,22,24,25^

The finding of short LOS in the Medicaid-only other race category is of interest but difficult to interpret. This category constitutes more than 10% of the Medicaid-only population, yet less than 1% of duals. It is possible that more people in this younger, more diverse population identify themselves as biracial or as having multiple race categories that are not captured by existing systems. Future research should seek a better understanding of who is in the other race category and aim for improved measurement of racial and ethnic identity overall.

In multivariable analyses, additional factors were associated with both outcomes. Female decedents were more likely to use hospice and less likely to have a short hospice LOS. Higher age was associated with hospice use for both groups and with lower odds of short hospice LOS only for duals. These findings may indicate that physicians and other clinicians more commonly refer women and older people to hospice, that older people and women are more receptive to referrals, or that disease severity and engagement with the health care system may vary by age or gender as well as race and ethnicity. State Medicaid policies and future research should consider why men and younger individuals are less likely to use hospice, and to use it for shorter periods.

It is noteworthy that only 32.1% of Medicaid-only decedents used hospice, much less than the roughly half of the Medicare population reported in previous Medicare research.^1,13^ They may wish to pursue more curative treatment for longer periods to extend their lives, and physicians may be more reluctant to recommend hospice for younger individuals. Of decedents who used hospice, both groups experienced short hospice LOS at a rate of about 40%, which is somewhat higher than the 33% to 35% found in previous research,^16^ suggesting room for improvement in referring all Medicaid recipients to hospice earlier in the course of terminal illness, when it can contribute to better quality of life. Eligibility criteria that require forgoing curative care to receive hospice contributes to short LOS,^26^ which may be a more salient factor for the Medicaid-only population.

Additional policy questions raised by these results include why the diverse, low-income population without Medicare are less likely to use hospice, and to what extent could improvements to clinician prognostication,^27^ individuals’ prognostic understanding,^28,29^ physician-patient communication, clinician attitudes toward hospice,^30^ or changes to hospice rules facilitate increased and longer hospice use. Policymakers would benefit from more research and intervention development to improve hospice use for younger, male, Black, and Hispanic people with Medicaid, and to enhance oversight of hospice care.^31^ More research on the non-Medicare hospice population is particularly needed because it is understudied and does not appear in most national reports relying on Medicare data.

One intriguing and novel finding concerns the likelihood of hospice use for individuals who had a nursing facility stay within 60 days of death. Among duals, decedents with a nursing facility stay were far less likely to use hospice (OR = 0.60) and more than twice as likely (OR = 2.62) to have a short hospice LOS. By contrast, Medicaid-only decedents with a nursing facility stay were 1.5 times more likely to have used hospice, although hospice LOS did not differ. It is unknown why duals with nursing facility stays were less likely to use hospice and more likely to have shorter hospice LOS. Further research is needed to understand barriers to and facilitators of hospice use among different Medicaid populations.

### Limitations

This study has several limitations. Data came from 1 state, and not all hospice-appropriate diagnoses were included. Although Connecticut’s hospice benefit tracks closely with the MHB, other states may have less generous hospice policies and may have different racial and ethnic populations.^9^ The first year of the COVID-19 pandemic was 2020, and although there was a slight decrease in Medicare decedents served by the MHB in 2020,^4^ and quality of hospice care may have suffered,^32^ the pandemic’s extended effects on hospice use are unknown. Because Medicare Advantage data were not available, only people with traditional Medicare were included. Historically, Medicare Advantage members reverted to traditional Medicare when they elected for hospice. Future research on Medicare Advantage and hospice will be important considering recent changes to, and experimentation with, the hospice carve-out, eg, the Medicare Advantage Value-Based Insurance Design Model.^33,34,35^ This study included only decedents and did not measure live discharges from hospice, another important outcome future studies could assess.^36^ As we used a limited number of confounders in our regression models, unobserved confounding due to other comorbidities is possible.

## Conclusions

This research analyzed use of hospice and LOS among a Medicaid population that has received limited attention in studies, particularly focusing on its diverse racial and ethnic composition. Findings raise concerns about equity of access and timing of access to hospice for the Medicaid population. Identified disparities call for additional investigation into the factors that affect referral to and acceptance of hospice care.

## Notes

Supplement. Data Sharing Statement.
