# Errors in the Abstract Results and Table

**DOI:** 10.1001/jamahealthforum.2023.5350

**Published:** 2024-01-26

In the Original Investigation titled “Racial and Ethnic Differences in Hospice Use Among Medicaid-Only and Dual-Eligible Decedents”^1^ published on December 8, 2023, there was a word missing in the first sentence of the Abstract Results. “Medicaid only” was added before 2407 in the first sentence. In addition, there was a 1 missing before the total number in the male sex row of the Table. This article was corrected online.
