# Errors in Table 2 and Table 4

**DOI:** 10.1001/jamahealthforum.2024.0409

**Published:** 2024-03-15

In the Original Investigation titled “Racial and Ethnic Differences in Hospice Use Among Medicaid-Only and Dual-Eligible Decedents”^1^ published on December 8, 2023, there were errors that appeared in Tables 2 and 4. There was an error in the number displayed in the Title of Table 2 and in Table 4 there was an error in the odds ratio reported for non-Hispanic Black participants. This article was corrected online.
