# Supplementary material for: Racial and Ethnic Differences in Hospice Use Among Medicaid-Only and Dual-Eligible Decedents
Source: JAMA Health Forum. 2023 Dec 8;4(12):e234240. doi: 10.1001/jamahealthforum.2023.4240 (PMC10709774; doi:10.1001/jamahealthforum.2023.4240)
Supplement: Supplement. — Data Sharing Statement. [file jamahealthforum-e234240-s001.pdf]

Data Sharing Statement

Robison. Racial and Ethnic Differences in Hospice Use Among Medicaid-Only and Dual-Eligible Decedents. *JAMA Health Forum*. Published December 08, 2023.
doi:10.1001/jamahealthforum.2023.4240

Data
Data available: No

Additional Information
Explanation for why data not available: These data include Medicare claims data and are therefore protected under a data use agreement from sharing.
